# Hospital and Institutional News

**Published:** 1913-08-09

**Authors:** 


### HOSPITAL AND INSTITUTIONAL NEWS.

### THE KING AT THE ROYAL WEST SUSSEX HOSPITAL.

We publish elsewhere this week a remarkable illustrated interview with Mr. Tom B. Harrison, the secretary of the Royal West Sussex Hospital, Chichester, which the King re-opened last Saturday, after the extensive alterations which we describe. The improvements and changes which His Majesty was able to inspect have brought the hospital up to date, and remedied certain serious defects which characterised the old construction. It must, therefore, have been exceedingly gratifying to Major Chauncy, the hospital's chairman, and to all sections of the staff, that the King's presence was possible at the re-opening after a long interval of interrupted work, and especially that the King, as a sign of his approval, has accorded the long desired style of Royal to the institution. Mrs. William James, whose late husband's generosity and interest played so important a part in the reconstruction scheme, was among those present, and the presentations included Lady Giffard, representing the executive staff of the hospital, Mrs. Garland, Mrs. Henty, and the Rev. Chancellor Davey. The King, who was accompanied by the Duke of Richmond and Gordon, arrived at the hospital from Goodwood, and after visiting the various departments, including the laundry, which was described in The Hospital a year ago, the new wards, and the new out-patient unit, signed the visitors' book and left for Cowes. The proceedings of last Saturday inaugurate a new period of development in the hospital's life, which it now begins under royal auspices and with royal approval; and we are sure that the whole staff, and especially perhaps the Matron, Miss E. I. E. Jones, on whom has fallen so much of the burden attendant on administering an institution for so long in the hands of the builders, and yet always far from closed, will be only too glad to settle down to steady routine work again. That this may be assured the public of Sussex has only to follow the King's example and support the new development which has been brought about so happily.

### WHO'S WHO AT THE MEDICAL CONGRESS.

At a time when London is entertaining the largest number of eminent medical men which has ever visited her at one time it becomes us to know something of the work as well as the fame of our distinguished visitors. Foreign Government delegates, special authorities—for once that word may be rescued from the lay papers—and readers of papers are mingled together. The official delegates of the United States comprise Professor Harvey Cushing, of Harvard University, whose work on the surgical treatment of tumours of the pituitary body has proved his latest, though not his only, title to fame. He is giving the address in surgery on Thursday this week. Sir William Osler, who presides over the Section of Medicine, needs no introduction to our readers who still have his recent warning as to the need for introducing pay wards into the voluntary system in their ears. Another delegate is Dr. Simon Flexner, of Johns Hopkins University, and the well-known Director of Laboratories at the Rockefeller Institute for Medical Research, whose researches into infantile paralysis and serum treatment of meningitis are respected equally in Europe and America. Professor Rudolph Matas, of New Orleans, is another American surgeon who has given his name to an operation for aneurism. "Arterial Surgery" will be the subject in his formal charge at the Congress. The thermal treatment, which is beginning to be adopted in this country, will have its advocate in Landouzy, the eminent delegate from France, and Switzerland is represented by Sahli, whose work on the ductless glands has brought him a European reputation.

### FAMOUS "REPORTERS" AND THEIR WORK.

The special authorities who have been chosen to open the discussions contain a specialist who has attracted attention only recently in a very different sphere from that of medicine. We refer, of course, to Dr. Paul Heger, professionally known as an honorary professor at Brussels University, but now to literature and the lay public as the donor of Charlotte Brontë's recently published letters to the British Museum. He is the son of "the professor" who suggested the hero of Charlotte Brontë's novel of that name. Another now semi-popular as well as famous name is that of Professor Ehrlich, of Frankfurt, the discoverer of "606" or salvarsan. Professor Verworn, of Bonn, who wrote the article on Physiology in the "Encyclopædia Britannica," reports at the Section on this subject. He is also known as the author of a novel theory on sleep. We must not forget those two protagonists of pathology, Dr. Crile and Professor Yandell Henderson, of America, to whose rival theories of surgical shock we have already referred. The address in medicine given on Wednesday was in the hands of Professor Chauffard, of Paris, and Dr. Freund, of Vienna, as well as Löffler, whose bacillus, the micro-organism of diphtheria, and whose work on stains are known everywhere, are taking part in the Section of Bacteriology. Professor Van Noorden, the reporter on diabetes; Professor Widal, of Paris, whose reaction has done so much to confirm the diagnosis of typhoid fever; Professor Brill, of New York, whose name is given to a fever as to which authorities are not yet agreed whether it is or is not a mild form of typhus; Professor Tuffier, of Paris, who reports on chest surgery; Dr. Russell Fowler, whose "position" has done so much for the recovery of patients after abdominal operations, are among the most prominent exponents of these branches of medicine and surgery. In the Section of Anæsthetics Dr. Fergusson, of New Jersey, will report on his open ether method. The combination of oxygen with nitrous oxide, which the lay public will always associate with a visit to the dentist, as another method of inducing anæsthesia will be reported by Dr. Teter, of Cleveland, U.S.A., who has developed the work of Sir Frederick Hewitt, anæsthetist to the King. The method of venous injection is reported by Professor Burckhardt, of Nuremberg.

### POPULAR DISCOVERERS IN VARIED SUBJECTS.

Elsewhere in this issue we have something to say about the Conference on Infant Mortality addressed by Mr. Burns on Monday. The reporter on this subject at the Congress is Dr. Henry Koplik, of New York, whose work in confirming the diagnosis of measles by his observation of certain spots within the mouth has to be mastered and remembered by every medical student. Curiously enough at the moment, there does not appear to be any conference on cancer, but Professor Wertheim, of Vienna, is discussing at the Congress the operative treatment which is named after him. The latest aids to diagnosis, so dearly loved by the general newspapers at holiday time, will be described in the Section of Laryngology by Professors Killian, of Berlin, and Chevalier Jackson, of Pittsburg, whose ingenuity has rendered many hitherto invisible cavities and positions in the body obvious to the human eye. The discoverer of the germ of malaria, Professor Laveran, once Army surgeon in Algiers, and scientific precursor of our own Sir Ronald Ross, will also be present. Indeed the galaxy is far from complete, and the above brief summary serves only to remind us of the honour done to London this week. It may possibly help, however, to direct attention to the speakers and readers of papers on the part of those who wish to know who is who in a particular Section.

### THE KERR MEMORIAL: A SUGGESTION.

The fund in memory of the late Father Kerr at Wimbledon has reached over £800, and at a recent meeting of subscribers it was decided that the sums collected should be handed over to the Trustees of the Nelson and Wimbledon Hospitals to be invested as an endowment fund in perpetuity, the interest to be used to endow a cot to Father Kerr's memory. The Assistant Honorary Treasurer of the fund, Mr. John Lyne, stated that while collecting he had heard a number of suggestions made, and there seemed to be a general feeling that the memorial should be in some public place where all passers-by could see it. This being so, we think that steps should be taken to satisfy the desire of the subscribers. Why could not a tablet with a bold inscription be placed on the outside wall of each hospital recording that "out of affection for Father Kerr the people of Wimbledon have endowed a cot to his memory in this institution"? As one gentleman has been found willing and generous enough to defray the expenses of the fund, two more surely could be found to pay the cost of the tablets here suggested.

### BY-WAYS OF RAISING MONEY.

In view of the widespread desire to raise funds for voluntarily supported institutions the figures issued by the committee of the annual "Woodford Meet of Cyclists" are of interest. The organisers among other methods of raising money use what they term "Hospital Stamps," which are receipts for a penny subscribed and which this year brought in some £132. 1,020 collecting boxes were issued and realised £373; and with collecting cards and subscriptions the sum of £600 was obtained, the highest amount yet taken from the annual meet. Perhaps it is worth while pointing out that this annual meet of cyclists is a function at which hundreds of cyclists come together and join a procession—many in fancy costumes—and parade the principal streets collecting on behalf of the local institutions.

### LEGACIES FOR A HOSPITAL STAFF.

We have already recorded the very large sums left by the will of the late Mr. Joseph Storrs Fry to several Bristol hospitals. Further particulars of a much more unusual kind are now announced, concerning nothing less than legacies to the staff and employees of the Bristol General Hospital as such; that is, quite apart from any personal relation between the testator and the legatees. Various amounts are bequeathed to certain officials and senior members of the female staff; these, we may presume, reflect direct knowledge and appreciation on Mr. Fry's part concerning the individuals concerned. Then, further, he directs that each of the other sisters serving the hospital at the time of his death shall get a legacy of ten pounds; and the same for each male employee of two years' service. One hundred pounds is to be divided among the female domestic servants and laundrymaids of two years' standing. In congratulating the beneficiaries on their good fortune, we can only add an expression of the good sense and good taste which led Mr. Fry to take these steps. Hospital sisters and employees generally very seldom receive such practical marks of appreciation from the public; and we trust that other testators may follow the example thus set them.

### AN OLD PATIENT'S GRATITUDE.

A striking, and to hospital workers most encouraging, instance of the good impression made upon patients by careful and considerate treatment in the wards is given in a recent number of the *Guy's Hospital Gazette*. The sister in charge of Naaman Ward, in a letter to the Editor, states that: "On Sunday last a working man was walking round Naaman Ward, and was asked what he wanted. He replied that in 1884 he had been a patient in the ward with a bad knee, and felt that he must come and look round. When he had gone a note of thanks and blessing to the hospital, with 2s., was left by him on the ward table." This return, after practically twenty years, is certainly a record. There are many cases in which patients have shown posthumous gratitude by legacies to institutions in which they have been treated, but comparatively few in which, after so long an absence, they have had the courage to prove their thankfulness by a personal visit and a contribution which probably means more than a hundredfold bigger legacy. There is one classical example of the patient whose leg had been amputated at a Continental hospital, and who twenty-five years later brought a thankoffering of pork chops to the young surgeon—then risen to be a master in his art—who had treated him. One advantage of having pay wards in a hospital is that they attract gifts of gratitude, sometimes on a large scale.

### "THE HOSPITAL'S" GUIDE TO CONGRESS VISITORS.

Whether visitors to the seventeenth International Congress of Medicine this week devote most of their time to inspecting the medical museums and libraries or the hospitals of London, the amount for them to see, or to choose from, is certainly overwhelming. A bird's-eye view of the hospitals alone would be of the greatest use if it combined brief information as to their history, chief treasures, and the best way of reaching them by train or omnibus. The Hospital, recognising the need, has done its best to supply it by publishing a delightfully printed Pocket-Guide to thirteen large hospitals of London, including King's College, with charming inset etchings of their chief architectural beauties. This is our free gift to all visitors to the International Congress of Medicine held at the Imperial Institute, and The Hospital's contribution to the convenience of the visitors. The Secretaries of the respective hospitals have made the Editor's task an easy one by ungrudgingly assisting in its compilation, and we believe they welcomed the opportunity of placing by means of their own newspaper a useful Guide in the hands of every visitor.

### A DOCTOR'S BEQUEST TO GUY'S HOSPITAL.

Mr. James Henry Targett, M.B., M.S., F.R.C.S., obstetric surgeon and lecturer at Guy's Hospital, and senior examiner in midwifery to the Conjoint Examining Board, left his shares in the Clinical Research Association, Limited, to the Treasurer of Guy's Hospital for the benefit of the pathological department of Guy's medical school. Mr. Targett, who died at the end of May, was only fifty-one at the time of his death. In spite of all his hard work, distinction, and apparent success his estate did not total more than £35,000. A successful lawyer, actor, or novelist would hardly prefer the rewards of medicine we think.

### HALIFAX HOSPITAL TO BE CLOSED.

The Eye, Ear, and Throat Hospital, Halifax, it has been decided at the annual meeting held last week, will be closed permanently at the end of this year. Mr. W. S. Milligan, who presided at the meeting, in moving that the institution be closed, said that it was now at the height of its prospe[illegible] but that circumstances made its closing necessary. When it was founded in 1886 there was a great need for it, and for several years Dr. John Oakley had supported it entirely, till later it relied for support mainly on the subscriptions of working men. Owing to the starting of an eye, ear, and throat department at the Royal Infirmary some years ago, he added, the two institutions had overlapped. Moreover, now that most working people were insured and could claim panel doctors, there was, he thought, another reason why the hospital should be closed. The main reason, however, was that Dr. Oakley and his son, Dr. Gardner Oakley, on whose honorary services the hospital was dependent, intended to resign at the end of the year. The institution could not afford to pay a resident medical officer, and there was not sufficient work for a full-time man to be employed. He hoped that subscribers would transfer their support to the Royal Infirmary. Dr. Oakley, in support of the motion, said that the building was unsuitable for a hospital, and, were the work to be carried on, there would have to be three wards instead of one, and that would mean an increased staff and prohibitive expense. He and his son had been economical, and had been surgeons, nurses, dispensers, and everything, and he did not think they would ever get that done again. With all due respect to their past efforts, we think it would be undesirable if it were.

### PAY OF WOMEN HOUSE PHYSICIANS.

The annual report of the board of management to the seventy-seventh anniversary Court of Governors of the East Suffolk and Ipswich Hospital is an unusually full and instructive document. As at so many kindred institutions, the section of finance reveals cause for serious anxiety. During the past year securities realising £3,480 had to be sold to meet current expenses; and not a single item to augment the capital account was received during the year. The total of investments now stands at a lower figure than at any time in the past ten years. Apart from this the record is one of progress and expansion, things desirable enough in themselves, but likely to create additional strain on the financial resources of the institution. Thus an extension of the out-patients' department has been under construction, and was opened recently just after the close of the financial year. The nurses' home is another department which has required and undergone extension; and a water-softening plant is yet a third new departure. The universal difficulty of securing resident medical officers led the board to raise the salaries offered; but even this expedient failed, so it was decided to accept a woman candidate for the post of house physician. A dental department has also been added to the pre-existing activities of the hospital. In view of all these increased responsibilities it is not surprising to learn that the nursing staff has had to be increased, to the extent of a deputy matron, an out-patient sister, and several nurses. Space forbids us to enter fully into the above appointments, but it is worth noting that the scarcity of residents is giving women doctors a further opening.

### THE TUBERCULIN ADVOCACY.

A sanatorium correspondent writes: "As an interested, though lay, member of the audience at the annual Conference of the National Association for the Prevention of Consumption during this week, one was much struck by the great diversity of medical opinion on the subject of tuberculin treatment, which occupied the first day's Conference. The speakers were mostly very decided in the notes they struck. A few were in favour of tuberculin as a routine of treatment for all forms of tuberculosis, some entirely against it, others again sounded warning notes on the need for very careful selection of cases, and others there were still who advocated its use in the sanatoria alone, where proper observation of the patient could be assured." We are not surprised that our correspondent has received the same opinion from the Conference as that which we have often laid down in these columns—namely, that treatment by tuberculin is still in the experimental stage. So long as there are so many different methods of carrying out this treatment, such varied doses, and such diverse prescriptions, each of which has a party vaunting its own method as a specific, the sick at large must be deprived of that residuum of good which has yet to be agreed upon in this branch of treatment. While doctors differ patients have to wait.

### STAFF CHANGES AT PARK FEVER HOSPITAL.

MANY changes are in progress at the Park Hospital. The senior assistant medical officer, Dr. Montagu L. Hine, having resigned with a view to specialising in ophthalmology, has left to take up the post of resident surgeon at the Westminster Ophthalmic Hospital. He will be succeeded by Dr. Taylor, late junior resident medical officer at the Lewisham Infirmary. Of the junior resident medical officers Dr. James Rae, known in medical literary circles as the author of the "Death of English Kings," leaves to take up duty at the London Fever Hospital, and Dr. R. P. Garrow leaves the children's for the fever service of the Metropolitan Asylums Board in September, while Dr. H. B. Morgan has been appointed senior house physician at the West Ham and Eastern General Hospital. Miss Villiers' successor as matron has not been appointed yet, and it is probable that nothing will be done till after the vacation. Such a complete change in the medical staff is not a common occurrence in institutional life, especially in the service of the Metropolitan Asylums Board.

### OFFICIAL INQUIRY INTO DISPENSER'S APPOINTMENT.

MR. J. S. OXLEY, on behalf of the Local Government Board, has held an inquiry into the circumstances attending the appointment of Miss Edith Mary Barrett as lady dispenser under the Poplar Guardians. The Guardians had presented a memorial to the Local Government Board following allegations that Mr. J. D. Hendry, one of the Guardians' medical officers, had offered money to two Guardians after her appointment. In February last, it appears, Miss Barrett exchanged a part-time appointment as dispenser at Blackwall local dispensary at a salary of £45 for a whole-time appointment as dispenser under the Guardians at a salary of £120. It was said that Dr. Hendry had interested himself in her election, and after which had offered money to Mr. F. T. Munnings and Mr. A. R. Adams, members of the Board, in consideration of having supported the lady. It was this allegation, the Guardians' legal representative pointed out, that had led the Board to seek an inquiry. Mr. Munnings, vice-chairman of the Board, gave evidence in support of the offer alluded to, as did Mr. Adams, the chairman. Dr. Hendry denied emphatically the offer of a bribe, reward, or recompense. Miss Barrett was his wife's cousin and he was anxious that she should get the appointment. Though he might have met and thanked the Guardians, there was, he said, not a word of truth in Mr. Munnings' account of the conversation. Miss Barrett denied knowledge of any reward being offered on her behalf to assist her in obtaining the post. The inquiry then closed, but it is difficult to see how the Poplar administration can escape censure however the result be declared. If the Guardians are right their officers include a type and show a spirit fatal to good administration. If the doctor is right, the officers are responsible to Guardians include a type equally undesirable.

### A HOSPITAL FRIEND OF FLORENCE NIGHTINGALE.

LADY ALICIA BLACKWOOD, one of the last surviving ladies who took a prominent part in the Crimea, died last week at the great age of ninety-four. So long ago as 1854 Lady Alicia and her husband arrived at Scutari and at once offered her help to Florence Nightingale and assisted in organising a hospital for the wives of the soldiers, several hundred of whom were herded literally like pigs in misery and dirt in the basement of the Scutari barrack hospital. Those who want the details of this story of tragi-comedy, for a comic incident or two is inevitable where human nature is faced with necessity of any description, should turn to her own account of these two years. All who have read it will not fail to be interested in the attempts made by Lady Alicia to rescue the women employed in the hospital laundry from the attractions of the local spirit—on sale everywhere—by opening a sort of penny bazaar at which the articles which their need really required, such as tea or flannel for clothing, and even headgear, could be purchased at truly "popular" prices. On her return to England Lady Alicia spent some years in London, where she published a series of drawings of Crimean landscape, and later retired to Boxmoor, where, though in seclusion from the world, she spent her still remaining and active energies in charitable works of varied kinds. In her one of the great ladies of the nineteenth century has passed away, a century which was rich in women personalities of such diverse kind as Florence Nightingale, George Eliot, and Mrs. Norton.

### THE GROWTH OF THE WOMAN SURGEON.

A RATHER striking comment on Professor Hochenegg's lately expressed opinions as to the unsuitability of the medical profession for women is the wide success which women students have gained at the recent examination for membership of the Royal College of Surgeons. Of course, it is possible to argue that success in examinations does not necessarily imply capacity for professional life in the same branch of study. Still it is noteworthy that so many new members of the Royal College of Surgeons should have been women; what light does this throw on their capacity for general practice?

### CONTROLLING THE MILK SUPPLY IN THEORY AND PRACTICE.

Readers of The Hospital will be in a better position than most people to appreciate the discussion at the Administrative Section of the Conference on Infant Welfare which was held last Tuesday, and at which the control of the milk supply was discussed. Dr. J. M. Beattie, Professor of Bacteriology in the University of Liverpool, discussed its administrative control with reference to tuberculosis, and said that all milk given to children should be sterilised, and that cows, sheds, and dairies should be compulsorily inspected. Assistant Surgeon-General J. W. Kerr, of the United States Public Health Service, read a paper on the Supervision of Milk in America, and said how much this contributed to its cleanliness. Now, inspection is all very well if it can be carried out, and the cost it entails is frankly accepted. But it is the details here which decide the value, as they mount up the cost, of everything. So we make no apology for referring those interested in this Section of the Conference on Infant Welfare to the series of articles which Dr. E. E. Norton, Tuberculosis Officer for Middlesex, has just concluded in our columns. He pointed out that contracts often read very nicely, but that their clauses were often far from being carried out, and he frankly faced a rise in the cost of milk to the public as a probability if the supervision of the milk supply were to be thorough. All who want really to understand this question, to see what it involves in practice, could not do better than to read Dr. Norton's detailed consideration in the recent back numbers of The Hospital.

### CAMBRIDGE'S WORK FOR DENTAL REFORM.

Among the congresses and meetings which are filling London and provincial centres with immense activity this summer, the annual meeting of the British Dental Association at Cambridge deserves more attention than perhaps it may receive. Professor Howard Marsh presided, and the presidential address was remarkable both as a demonstration and a reminder. Twenty-eight years ago, the President remarked, the Association met in Cambridge, and he only hoped the result of their deliberations this year might be as practically important as they were then. A paper, which attracted little, if any, attention at that time was read by Mr. William Fisher on "Compulsory Attention to the Teeth of School Children." The resultant campaign, he added, they all knew. He then outlined the experiment at Cambridge, which Mr. Sedley Taylor's generosity had started, of a dental institute for school children. When the experiment was begun—the figures are instructive—94.8 per cent. of children were found to have carious teeth, but of the 2,225 children who had grown up with the scheme from five years of age, he said, only 29 were found to have unsaveable permanent teeth. The opposition of the parents was breaking down, and the President concluded his speech with a plea for a public dental service. The day of the dental clinic for school children is dawning, and the part played by dental disease in causing degeneration of innumerable kinds is being recognised. At a later meeting Mr. Vernon Knowles gave details of the public dental service which Reading set on foot in January last. The public, however, will require more explanation, we fear, of the functions of a public dental service, which Professor Howard Marsh would be doing good service to expound. If he does so, perhaps his hope will be realised that Cambridge which inaugurated the dental clinic should also prove the source of a public dental service. This could hardly encounter more opposition than the dental clinic did at first, and people now are more likely to be open to conviction.

### STAFF CHANGES AT THE LONDON HOSPITAL.

Dr. Francis Warner, F.R.C.P., who has been on the staff of the London Hospital since 1879, has resigned the post of senior physician. He will still, however, interview candidates for the nursing staff and will lecture on the mental development of weak-minded children. A new appointment has been created to the heart department of the London Hospital, that of special assistant, to which Dr. John Parkinson, a former registrar, has been appointed.

### THE HOUSE OF LORDS AND WESTMINSTER HOSPITAL REMOVAL BILL.

The Bill authorising the Governors of Westminster Hospital to build a new institution on land to be acquired in the County of London and to sell the existing site was allowed to proceed by the Committee of the House of Lords, presided over by the Duke of Wellington last week, with the insertion of a clause asked for by the Westminster Council. This clause provides that nothing in the Bill should affect their powers under the Paving Act of 1817 to make compulsory purchase of land for public improvements. Mr. Squarey, K.C., for the promoters of the Bill, outlined the history of the hospital's sites and recalled, as it is interesting to note to-day, that £6,000 was the purchase money of the present site in Broad Sanctuary in 1832. The Governors ask to be released from the restriction in their covenant which prevents the trustees from using the land for any other purpose than that of a hospital. It is satisfactory to note that in the event of a new building being erected on the site, Broad Sanctuary and Princes Street will be seven feet wider. Mr. Vaux Graham, vice-chairman, gave evidence that the site was one of the most valuable in London.

### FOREIGN ORTHOPÆDISTS IN LONDON.

An encouraging sign of the times is the dinner which Lord Denbigh, the committee, and the staff of the Royal National Orthopædic Hospital in Great Portland Street are giving at the hospital to the Orthopædic Sub-section of the International Medical Congress. How slow England was to appreciate the importance of this branch of surgery and to catch up with the experience and practice which have so markedly taken place on the Continent may be judged from the fact that the Royal National Orthopædic Hospital was opened, in an up-to-date sense, only four years ago, and that orthopædics as a special and seriously equipped department is still a rare feature of the big hospitals. The English, however, have a way of lagging behind other nations, and then of absorbing the results of their initiative so as to get ahead of them in the long run. We doubt if this is true yet of orthopædics, but it may very easily become true if full advantage is taken of the presence in England of such men as Professor Vulpius, of Heidelberg; Professor Dollinger, of Budapest; Professor Hans Spitzy, of Vienna; Dr. Redard, of Paris; to say nothing of the representatives of New York and Boston, who will all be present at the Orthopædic Hospital's dinner.

### PAYMENTS TO NON-PANEL DOCTORS.

At an inquest held at Islington on the body of a widow aged sixty-seven, who died suddenly of apoplexy, a witness stated that medical aid was unobtainable for six hours on a particular evening, since all the panel doctors were out. A panel doctor, Dr. Brown, however, declared that he was at home during his panel hours, 7 to 9, and that he received no call to see the deceased. The subject might then have dropped had not the Coroner asked whether the insured patient would have to pay the fee of another medical man if he could not obtain a panel doctor in case of emergency. Dr. Brown replied that the London Insurance Committee were considering a similar arrangement to one which prevailed at West Ham, whereby, if a panel doctor were out, another medical man could be called upon to attend free of cost to the patient. We understand that the payment of non-panel doctors under the suggested circumstances would be in the hands of the local Insurance Committee, which may decide to make any arrangements that it likes. If so it seems highly probable that the patient would be held responsible, though West Ham apparently has found a convenient solution of the difficulty. In this connection we may note that a somewhat similar difficulty has arisen in getting prescriptions made up on chemists' early closing days. Local Insurance Committees are being asked to provide for the dispensing of urgent prescriptions.

### VOLUNTARY EFFORT AND INFANT MORTALITY.

Mr. Frank Stokes, Secretary of South Marylebone General Dispensary, in pointing out the difficulties in which his institution is placed by the success of its work and the aid it has given to panel patients, draws attention to facts which might well be better recognised. In return—he says in effect—for being the pioneer institution to establish infant consultations in Great Britain, and for treating panel patients, for which others have received payment, and for treating tuberculosis patients at a cost of about £2 per case, more work is likely to be thrown upon it, while neither the money allotted for tuberculosis benefit, nor subscriptions from municipalities are forthcoming. The Local Government Board is advising municipalities to make use of the movement which the St. Marylebone General Dispensary has set on foot, but it does not appear to advise them to subscribe to it. Mr. Stokes finally points out that Boards of Guardians have power to assist institutions which relieve the poor-rate, and infant consultations have done so much to prevent infant mortality that the voluntary effort represented by these dispensaries, here as elsewhere, should be linked up with public bodies which are reaping the benefits that the voluntary system has provided.

### EXTRAORDINARY DIETS FOR INVALIDS.

Dr. E. G. Annis, medical officer of health for Greenwich, deals in his annual report with the prevalency of summer diarrhœa and the extraordinary diets given to children. During last summer the Borough Council had the services of a temporary health visitor, and Dr. Annis states that it was doubtless owing to her visitations that the number of deaths from diarrhœa was reduced. Many strange diets were noted by her, one of the most unsuitable for young children being what are locally known as "toffee apples." They are small apples, most probably fallings, and therefore in an unripe condition, either partially cooked or not cooked at all, which are dipped in syrup and sold at many of the small shops of the borough for three or four a penny. These, adds Dr. Annis, are luxuries much appreciated by children, and the health visitor frequently found the baby of only two or three months old being regaled with them. After such a diet one could naturally expect a severe attack of diarrhœa or convulsions. This year the visitation of children has been undertaken by the Ranyard Nurses' Society.

### THIS WEEK'S DRUG MARKET.

The Bank Holiday naturally interfered with the Drug market, but business is by no means dull and a good deal of interest is being taken in several articles. It is now definitely known that the agreement between the cinchona bark growers in Java and the European quinine manufacturers has been signed. The immediate result has been a small advance in the price of quinine and it is confidently anticipated that further advances will take place in due course, but it is not thought probable that large additions will be made to the price immediately; it would probably be wise to buy quinine at an early moment. The position of opium shows very little change, and although a large crop is expected, prices are fairly well maintained. Buyers, however, do not seem anxious to come forward as they are expecting lower prices before long. Morphine has been slightly reduced in price. The price of citric acid is well maintained and all the citrates are dearer. Cocaine seems to be tending dearer.

